# Dried Yeast Extracts Curtails Pulmonary Oxidative Stress, Inflammation and Tissue Destruction in a Model of Experimental Emphysema

**DOI:** 10.3390/antiox8090349

**Published:** 2019-09-01

**Authors:** Yun-Ho Kim, Min-Kyung Kang, Eun-Jung Lee, Dong Yeon Kim, Hyeongjoo Oh, Soo-Il Kim, Su Yeon Oh, Kyung-Hee Kim, Sang-Jae Park, Yean-Jung Choi, Young-Hee Kang

**Affiliations:** 1 The Korean Institute of Nutrition, Department of Food and Nutrition, Hallym University, Chuncheon 24252, Korea; 2 Mediense Co. Ltd., Chuncheon 24232, Korea; 3 Department of Bio-Food Science & Technology, Far East University, Eumseong 27601, Korea

**Keywords:** airway inflammation, cigarette smoke, dried yeast extracts, ovalbumin, oxidative stress, pulmonary emphysema

## Abstract

Pulmonary emphysema is characterized by a loss of alveolar integrity due to prolonged cigarette smoking and inhaled irritants. Dried yeast extracts (YE) are employed as food additives, savory flavorings, or creation of umami taste sensations. Despite being rich in nutrition, their application as nutraceuticals and functional foods is not investigated much and little is known about the inhibition of pulmonary emphysema. This study examined whether YE ameliorated pulmonary emphysema in mice is evoked by cigarette smoke (CS) and ovalbumin (OVA). Mice were orally administrated with 25–100 mg/kg YE for 8 weeks. Alveolar epithelial A549 cells exposed to lipopolysaccharide or CS extracts (CSE) were supplemented with 10–100 µg/mL YE. Oral YE administration reduced bronchoalveolar lavage fluid leukocytosis in CS-/OVA-exposed mice. YE reduced induction of inflammatory mediators and MMP-12, and diminished reactive oxygen species production and emphysematous alterations in CS-challenged airways. The YE treatment blunted bax/bcl-2 ratio and activation of p53 and caspases in CS-exposed lungs. Apoptotic death was dampened in CSE-loaded YE-supplemented A549 cells. YE curtailed tissue levels of MMP-12 in inflammatory OVA-exposed lungs. YE abrogated the secretion of TNF-α and MCP-1 through blocking NF-κB signaling in endotoxin-loaded A549 cells. Thus, the antioxidant YE may therapeutically ameliorate oxidative stress and inflammatory tissue destruction in emphysematous diseases.

## 1. Introduction

Chronic obstructive pulmonary disease (COPD) is a chronic inflammatory lung disease with the resultant airflow obstruction from the lungs [1,2]. Its symptoms include breathing difficulty, cough, mucus (sputum) production, and wheezing [3,4]. Chronic bronchitis and emphysema are the most common disorders responsible for COPD, being caused by long-term exposure to irritating noxious gases, most often from cigarette smoke (CS) [5]. Chronic bronchitis is inflammation of the lining of the bronchial tubes, and emphysema is a condition in which the alveoli of the lungs are destroyed [6]. In fact, pathological alterations in bronchioles and alveoli lead to a loss of alveolar integrity through activating aberrant inflammatory pathways [5]. Bronchiolar and alveolar epithelial cells can display direct immune and anti-inflammatory responses against lung tissue damage [6,7]. Pulmonary mesenchymal cells such as airway smooth muscle cells and lung fibroblasts can also respond to inflammatory mediators [6,8]. In COPD, chronic inflammation mainly entails the infiltration of neutrophils, macrophages, CD8+ T lymphocytes, and other inflammatory cells into the small airways [1,6]. Bronchiolar epithelium-derived monocyte chemoattractant protein (MCP)-1 and interleukin (IL)-8 can be responsible for the chemotactic activity of neutrophils [7,9]. However, the underlying mechanisms for bronchiolar and alveolar inflammation of COPD remain to be solved. Promising mechanisms involved in small airway/alveolar destruction and structural changes may provide major therapeutic targets in COPD [10,11].

Numerous studies have identified diverse therapeutic targets in chronic bronchitis and COPD [11,12]. The major therapeutic option for these diseases is to combat airway inflammation [12,13]. Exposure to CS provokes the recruitment of inflammatory cells into the airways and prompts immune response mechanisms [14]. Thus, the immunomodulatory therapies in airways may effectively alleviate pulmonary diseases [15]. The pathogenesis of emphysema refers to alveolar destruction with airspace enlargement and loss of alveolar integrity by the interaction of apoptosis, oxidative stress, and protease/antiprotease imbalance [14,16]. Oxidative stress is a purported contributor for emphysema through activating pro-inflammatory cytokine transcription [13,17]. The protease/antiprotease imbalance impairs tissues in COPD and emphysema, which is involved in inflammatory processes [17,18]. There are major novel anti-inflammatory agents in COPD targeting lung and systemic inflammation including inhaled corticosteroids and β-adrenergic receptor agonists, phosphodiesterase-4 inhibitors, macrolides, and statins [14,19,20]. These approaches can affect more intimately COPD-specific mechanisms of inflammation, mucin production, and tissue destruction and repair [14,16,19]. Currently, such treatments in COPD are not yet justified. Therefore, new therapeutic strategies with natural agents have mostly proven to be safe, and are currently under development for treating airway inflammation [21].

Several studies show potential roles of natural plant extracts and compounds for the treatment of asthma and COPD [22,23]. Anti-inflammatory and antioxidant polyphenols including resveratrol, curcumin, quercetin, sulforaphane, lycopene, mangiferin, and dihydroquercetin suppress experimental pulmonary fibrosis and modulate various biochemical features of COPD [24,25]. A MORGEN study suggests a beneficial effect of a high intake of catechins and solid fruits with flavonols and flavones against COPD [26]. Our previous studies revealed that several compounds such astragalin, kaempferol, and oleuropein antagonized airway epithelial apoptosis and fibrosis, Inflammation and airway thickening in ovalbumin (OVA)-challenged mice [27,28,29]. Additionally, oleuropein rich in olives exhibits favorable effects on pulmonary inflammation in CS-challenged mice through blocking recruitment of inflammatory and allergic cells and blunting alveolar destruction [29]. A recent study has reported that dried yeast extracts (YE) may effectively inhibit oxidative stress-responsive epithelial eosinophilia and mucus-secreting goblet cell hyperplasia in asthma [30]. However, the beneficial effects of YE on smoking-induced inflammation and emphysema in bronchioles and alveoli have not been reported. This study examined whether YE abrogated airway inflammation and apoptotic emphysema in CS- and OVA-challenged mouse models. Pulmonary inflammation and emphysema were also examined in alveolar epithelial cells exposed to cigarette smoke extract (CSE) and lipopolysaccharide (LPS).

## 2. Materials and Methods

### 2.1. Chemicals

RPMI, chicken egg white albumin, and LPS were obtained from the Sigma-Aldrich Chemical (St. Louis, MO, USA), as were all other reagents, unless specifically stated elsewhere. Fetal bovine serum (FBS), penicillin-streptomycin, and trypsin-EDTA were purchased from the Lonza (Walkersville, MD, USA). Rabbit polyclonal antibodies of matrix metalloproteinase (MMP)-12 and intracellular adhesion molecule (ICAM)-1, goat polyclonal cyclooxygenase (COX)-2 antibody, and mouse monoclonal inducible NOS (iNOS) antibody were purchased from the Santa Cruz Biotechnology (Dallas, TX, USA). Mouse monoclonal antibodies of bcl-2 and bax were provided by BD Transduction Laboratories (Franklin Lakes, NJ, USA). Rabbit polyclonal antibodies of cleaved caspase-3, cleaved caspase-9, phospho-p53, inhibitory κB (IκB), and phospho-IκB were obtained from Cell Signaling Technology (Beverly, MA, USA). Horseradish peroxidase (HRP)-conjugated goat anti-rabbit IgG, donkey anti-goat IgG, and goat anti-mouse IgG were purchased from Jackson Immuno-Research Laboratories (West Grove, PA, USA). Mouse monoclonal β-actin antibody was obtained from Sigma-Aldrich Chemicals. Essential fatty acid free bovine serum albumin (BSA) and skim milk were supplied by Becton Dickinson Company (Sparks, MD, USA). 4’,6-Diamidino-2-phenylindole (DAPI) was obtained from Santa Cruz Biotechnology.

### 2.2. Preparation of YE

YE used in the current study was provided by the Mediense Co. Ltd (Chuncheon, Korea). Dried yeast was extracted in boiled distilled water (10% *w/v*) at 40–60 °C for 24 h, followed by centrifugation at 3000 rpm for 10 min. The resulted supernatants were harvested after filtration with 0.45 μm. YE was dissolved in dimethyl sulfoxide (DMSO) for live culture with cells; a final culture concentration of DMSO was <0.5%.

### 2.3. Animal Experiments

Six week-old male BALB/c mice (Hallym University Breeding Center for Laboratory Animals) were used in this study. Female mice were excluded because of concerns that female hormone cycles would affect experiments. Mice were kept on a 12 h light/12 h dark cycle at 23 ± 1 °C with 50% ± 10% relative humidity under specific pathogen-free circumstances, fed a non-purified diet, and provided with water ad libitum at the animal facility of Hallym University. The present study was approved by the Hallym University Institutional Review Board and Committee on Animal Experimentation (Hallym 2017-56). This study was conducted in compliance with the University’s Guidelines for the Care and Use of Laboratory Animals.

Mice were acclimatized for 1 week before beginning the experiments. All mice were distributed among five subgroups (*n* = 9–10 for each subgroup).

Passive smoking models: Mice receiving the smoke challenge were further divided into four subgroups. One subgroup (no CS) did not receive the smoke challenge. YE solution (containing 25–100 mg/kg BW) was orally administrated to mice 1 h via oral gavage once a day (5 days/week) for 8 weeks before. Subsequently, mice were exposed to smoke of research cigarettes (11 mg tar and 0.7 mg nicotine/cigarette) for 30 min in a specially designed chamber once a day for 8 weeks. Research cigarettes (3R4F, 11 mg tar and 0.7 mg nicotine per cigarette) were obtained from the University of Kentucky (Lexington, KY, USA).

Mouse asthma model: Mice receiving the OVA challenge were further divided into five subgroups. One subgroup (no OVA) did not receive the OVA challenge. Mice were sensitized with 20 μg OVA dissolved in 30 μL phosphate buffered saline (PBS) with 50 μL Imject Alum (Thermo Fisher Scientific, Rockford, IL, USA) through subcutaneous injections on the days 0 and 14. Subsequently, 0.1 mL YE solution (containing 25–100 mg/kg BW) was administered via oral gavage to OVA-sensitized mice 1 h before challenge. On the days of 28−30, 5% OVA inhalation to mice was carried out for 20 min in a plastic chamber linked to an ultrasonic nebulizer (Clenny2 Aerosol, Medel, Italy). Control mice were sensitized and challenged with PBS as the OVA vehicle. All mice were sacrificed 24 h after the latest provocation (day 30).

All the mice were killed with an anesthetic dose of 0.3 g/kg avertin and 8 μg/kg *tert*-amyl alcohol. The trachea was cannulated, and both lungs and airways were rinsed in 1 ml PBS for the collection of bronchoalveolar lavage fluid (BALF). The numbers of inflammatory cells including neutrophils and eosinophils in BALF were determined using a Hemavet HV950 Multispecies Hematologic Analyzer (Drew Scientific, Oxford, CT, USA). The right lungs were collected, frozen in liquid nitrogen, and kept at −80 °C until used for Western blotting. Left lungs were preserved and fixed in 4% paraformaldehyde and then used for immunohistochemical analyses.

### 2.4. Preparation of CSE for Cell Culture

The research cigarettes were consecutively smoked through an experimental apparatus with a constant airflow driven by an air vacuum pump. The collected CS was bubbled in 10 mL PBS. The resulting smoke suspension was filtered through a 0.22 μm pore filter in order to eliminate bacteria and large particles. The filtrates referred to a 100% CSE.

### 2.5. A549 Cell Culture and Viability

Human alveolar basal epithelial cells A549 cells were provided by the American Type Culture Collection (Manassas, VA, USA). A549 cells were cultured in RPMI 1640 supplemented with 10% FBS, 2 mM l-glutamine, 100 U/mL penicillin, and 100 μg/mL streptomycin. A549 cells were sustained in 90–95% confluence at 37 °C in an atmosphere of 5% CO_2_. A549 cells were treated with 10–100 μg/mL YE and then stimulated with 2 μg/mL LPS or 5% CSE for up to 24 h to induce expression of target gene proteins. 

The cytotoxicity of YE was determined using 3-(4,5-dimetylthiazol-yl)-diphenyl tetrazolium bromide (MTT, Duchefa Biochemie, Haarlem, Netherlands) after culture of A549 cells. These cells were incubated in a fresh medium containing 1 mg/mL MTT for 3 h at 37 °C. The purple formazan product was dissolved in 0.5 mL isopropanol with gentle shaking. Absorbance of formazan was measured at λ = 570 nm using a microplate reader (Bio-Rad Model 550, Hercules, CA, USA). 

### 2.6. Staining with Hematoxylin and Eosin (H&E)

For the histological analyses of airways, small airway and alveolar specimens provided at the end of the experiments were fixed in 10% paraformaldehyde. The paraffin-embedded specimens were sectioned at 5 μm thickness, deparaffinized and stained with hematoxylin and eosin (H&E) stain for 2 min, and quickly dehydrated in 95% absolute alcohol. The H&E-stained tissue sections were observed using an optical microscope Axioimager system equipped for fluorescence illumination (Zeiss, Gottingen, Germany). Five images were taken from each tissue section.

### 2.7. Western Blot Analysis

Mouse lung tissue extracts and A549 cell lysates were prepared in 1 mM Tris-HCl (pH 6.8) lysis buffer containing 10% sodium dodecyl sulfate (SDS), 1% glycerophosphate, 0.1 mM Na_3_VO_4_, 0.5 mM NaF, and a protease inhibitor cocktail. Tissue extracts and cell lysates containing equal amounts of proteins were electrophoresed on 8%–15% SDS-PAGE and transferred onto a nitrocellulose membrane. Blocking a nonspecific binding was performed using either 3% fatty acid-free BSA or 5% non-fat dry skim milk for 3 h. The membrane was incubated overnight at 4 °C with a specific primary antibody of COX-2, iNOS or ICAM-1, bcl-2, bax, cleaved caspases, MMP-12, or IκB. The membrane was then applied to a secondary antibody conjugated to HRP for 1 h. Following triple washing, the target proteins were determined using the Immobilon Western Chemiluminescent HRP substrate (Millipore Corp., Billerica, MA, USA) and the Agfa medical X-ray film blue (Agfa HealthCare NV, Mortsel, Belgium). Incubation with β-actin antibody was conducted for the comparative control.

### 2.8. Immunohistochemical Staining

Paraffin-embedded tissue sections (5 μm thick) of small airways and alveoli were deparaffinized and hydrated in order to conduct immunofluorescent histochemical analyses. The sections were preincubated in a boiling sodium citrate buffer (10 mM sodium citrate, 0.05% Tween 20, pH 6.0) for the antigen retrieval. The tissues were blocked with 5% BSA in PBS for 1 h. A specific primary antibody against MMP-12 was incubated overnight with the sectioned tissues. Subsequently, the tissue sections were incubated for 1 h with fluorescein isothiocyanate-conjugated or Cy3-conjugated anti-rabbit IgG. For identification of nuclei, the fluorescent nucleic acid dye of DAPI was applied for 10 min. Stained tissues were mounted on slides using mounting medium (Vector Laboratories, Burlingame, CA, USA). Images of each slide were obtained with an optical microscope Axioimager system (Zeiss).

### 2.9. Dihydroethidium (DHE) Staining for Reactive Oxygen Species (ROS) Production

Paraffin-embedded tissue sections (5 μm thickness) of airways were deparaffinized and hydrated for the DHE staining. Airway tissues were stained by incubating for 1 h in 20 μM DHE (Invitrogen, Carlsbad, CA, USA). For the identification of nuclei, DAPI was given for 10 min. Stained tissues on slides were mounted in mounting solution. Images of each slide were taken using an optical microscope Axioimager system.

### 2.10. Hoechst 33258 Staining

The A549 cells were plated on a 24-well glass slide and incubated for 24 h in media containing 5% CSE in the absence or presence of 10–100 μg/mL YE. After the fixation of A549 cells with ice-cold 4% formaldehyde for 1 h on a glass slides, the nuclear stain Hoechst 33258 (Promega Co., Madison, WI, USA) was added at a final concentration of 10 μg/mL for 15 min to allow uptake and equilibration before microscopic observation. The slides were mounted while wet in aqueous VectaMount mounting solution. Cells containing fragmented or condensed nuclei were considered apoptotic. Images of each slide were taken using an optical microscope system.

### 2.11. Terminal Deoxynucleotidyl Transferase dUTP Nick End Labeling (TUNEL) Assay

The transferase dUTP nick end labeling (TUNEL) assay is a common method for detecting DNA fragments. The TUNEL assay was conducted using a commercial fluorometric TUNEL kit (Promega Co., Madison, WI, USA). The A549 cells were plated on a 24-well glass slide and incubated for 24 h in media containing 5% CSE in the absence or presence of 10–100 μg/mL YE. Cells fixed with ice-cold 4% formaldehyde for 20 min were permeabilized with 0.2% Triton X-100, and fragmented DNA was labeled with fluorescein-dUTP at 37 °C for 1 h. DAPI was used for counterstaining nuclei, and cells were visualized with an Axiomager optical microscope system.

### 2.12. Enzyme-Linked Immunosorbent Assay (ELISA)

Following culture protocols, the secretion of tumor necrosis factor (TNF)-α and MCP-1 in A549 cells was determined in collected culture medium supernatants by using ELISA kits (R&D Systems, Minneapolis, MN, USA), according to the manufacturer’s instructions.

### 2.13. Immunocytochemical Staining

A549 (7 × 10^4^ cells) grown on 24-well glass slides were exposed to LPS in the absence and presence of 10–100 μg/mL YE. A549 cells were fixed with 4% formaldehyde for 10 min and permeated with 0.1% Triton X-100 for 5 min on ice. Cells were blocked using a 4% FBS for 1 h. Immunofluorescent cytochemical staining of A549 cells was performed using NF-κB p50 antibody and Cy3-conjugated anti-rabbit IgG. Nuclear staining was performed with DAPI. Each slide was mounted in a VectaMount mounting medium and images were taken using an optical Axiomager microscope system. 

### 2.14. Statistical Analysis

The results were expressed as mean ± SEM for each treatment group in each experiment. Statistical analyses were performed using the Statistical Analysis Systems statistical software package (SAS Institute, Cary, NC, USA). Significance was determined by a one-way analysis of variance, followed by a Duncan range test for multiple comparisons. Differences were considered significant at *p* < 0.05.

## 3. Results

### 3.1. Inhibition of Airway Inflammation of CS-Exposed Airways by YE

**Figure 1:**
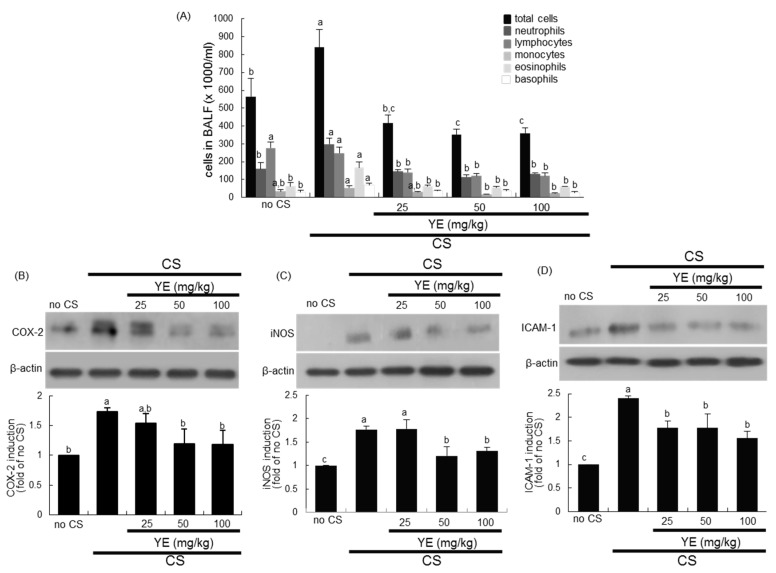
Leukocytes in the bronchoalveolar lavage fluid (BALF; **A**) and inhibition of pulmonary inflammation (**B**–**D**) in cigarette smoke (CS)-challenged mouse lungs treated with dried yeast extracts (YE). Mice were orally administrated with 25–100 mg/kg YE and exposed to CS for 30 min. Cells in BALF were counted using a Hemavet HV950 Multispecies Hematologic Analyzer (**A**). Tissue extracts were subject to Western blot analysis with a primary antibody against COX-2, iNOS, or ICAM-1. β-Actin protein was used as an internal control. The bottom bar graphs represent quantitative results of the upper bands obtained from a densitometer. Values (mean ± SEM, *n* = 3–4) in respective bar graphs not sharing a same small letter indicate a significant difference at *p* < 0.05.

This study examined how YE inhibited CS-evoked inflammation in mouse airways. Exposure of mice to CS increased total leukocyte number in the BALF by ≈1.5-fold (Figure 1A). Surprisingly, the challenge of CS promoted the influx of neutrophils and eosinophils in the BALF, indicating that CS resulted in neutrophilic and eosinophilic inflammation (Figure 1A). Oral administration of ≥25 mg/kg YE reduced CS-induced leukocytosis of neutrophils and eosinophils, which was incomparable to that of control mice (Figure 1A). In addition, YE curtailed the number of lymphocytes and monocytes in the BALF elevated in CS-exposed mice.

It was examined whether CS-stimulated airway inflammation was attenuated in YE-supplemented mice. The tissue level of COX-2 responsible for prostaglandin biosynthesis and inflammation was elevated in CS-exposed mouse lungs (Figure 1B). In addition, the airway tissue levels of iNOS and ICAM-1 directly involved in inflammatory responses were enhanced in CS-challenged mice (Figure 1C,D). However, orally-administrated YE reduced the induction of these inflammatory proteins promoted by exposure of airways to CS (Figure 1B–D).

### 3.2. Blockade of Emphysematous Injury of CS-Challenged Airways by YE

**Figure 2:**
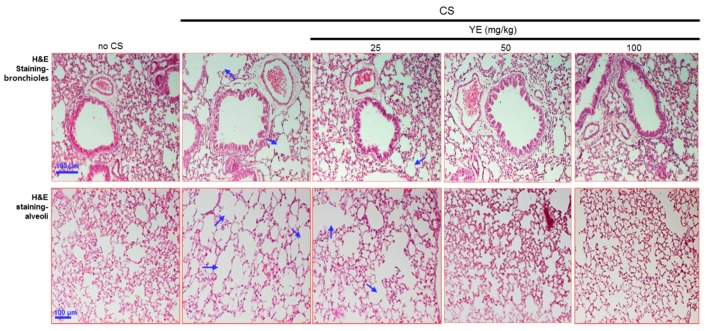
Blockade of airway destruction by dried yeast extracts (YE) in cigarette smoke (CS)-challenged mouse bronchioles and alveoli. Mice were orally administrated with 25–100 mg/kg YE and exposed to CS for 30 min. Airway tissue sections were stained by using a hematoxylin and eosin (H&E) reagent. Each photograph is representative of four mice. The arrows indicate damaged bronchioles and alveolar air sacs. Scale bars = 100 μm.

**Figure 3:**
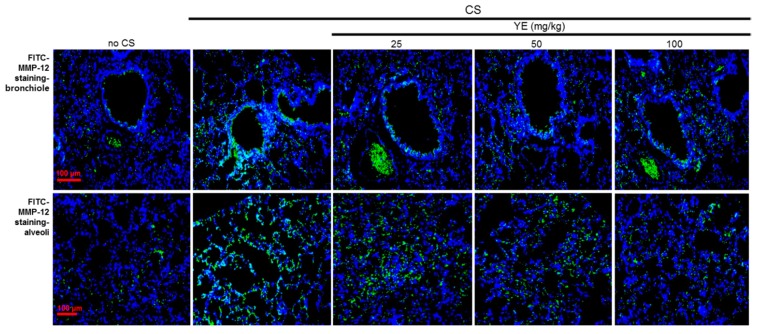
Inhibition of alveolar emphysema by dried yeast extracts (YE) in cigarette smoke (CS)-challenged mouse alveoli. Immunohistofluorescence analysis was done in tissues of small airways and alveoli of CS-challenged mice. The MMP-12 localization was identified as FITC-green staining in mouse airways exposed to CS. Nuclear staining was done with DAPI (blue). Each photograph is representative of four mice. Scale bars = 100 μm.

The histological examination was conducted in the lung tissues stained with H&E. The CS exposure induced the airway wall damage in mice (Figure 2). However, oral administration of 25–100 mg/kg YE highly attenuated the pathological alterations observed in bronchiolar and alveolar tissues of CS-challenged mice. In addition, MMP-12 was highly expressed in mouse airways exposed to CS, evidenced by FITC-immunostaining (Figure 3). In contrast, the treatment with 25–100 mg/kg YE reduced its induction in CS-exposed bronchioles (Figure 2). Moreover, the H&E histological staining revealed that the CS challenge to mice evoked destruction in pulmonary alveoli (Figure 2). Consistently, the MMP-12 induction was markedly enhanced. However, 25–100 mg/kg YE diminished the emphysematous damage in pulmonary alveoli and curtailed the FITC-staining of MMP-12 (Figure 3). Therefore, YE may inhibit emphysema and alveolar cell loss in CS-exposed small airways and alveoli. 

### 3.3. Inhibitory Effects of YE on CS-Induced Pulmonary Apoptosis and Oxidative Stress

**Figure 4:**
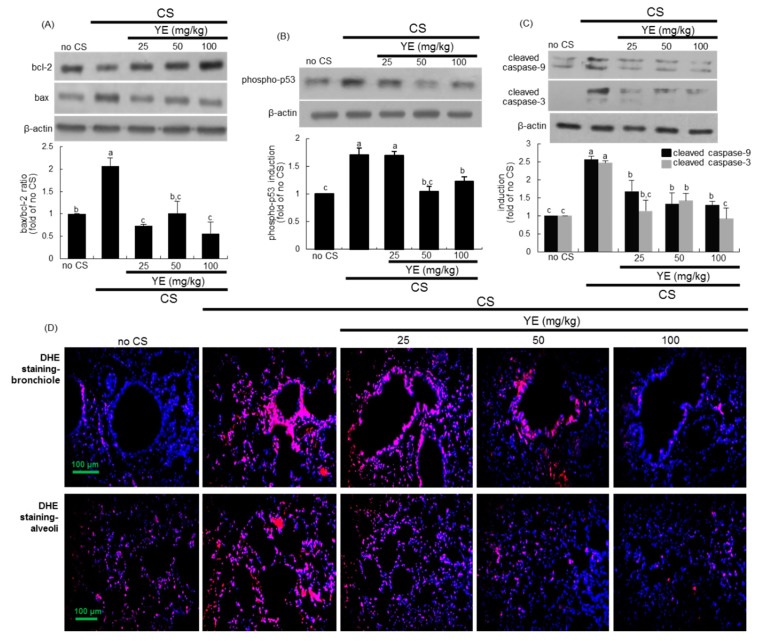
Inhibition of apoptotic lung injury and reactive oxygen species (ROS) production by 25–100 mg/kg yeast extracts (YE) in cigarette smoke (CS)-challenged mouse lungs. Tissue extracts were subject to Western blot with a primary antibody against bcl-2, bax, phospho-p53, cleaved caspase-9, or cleaved caspase-3 (**A**–**C**). β-Actin protein was used as an internal control. The bar graphs (mean ± SEM, *n* = 3) represent quantitative results of the upper bands obtained from a densitometer. Values in respective bar graphs not sharing a same small letter indicate a significant difference at *p* < 0.05. Dihydroethidium (DHE) staining showing pulmonary ROS production (**D**). Tissue sections of small airways and alveoli were stained with DHE, and nuclear staining was done with DAPI (blue). Each photograph is representative of four mice. Scale bars = 100 μm.

This study attempted to examine whether YE inhibited emphysematous airway damage through blocking pulmonary apoptosis and oxidative stress induced by CS. Western blot analysis showed that CS diminished the lung tissue level of anti-apoptotic bcl-2 and increased the level of pro-apoptotic bax, consequently elevating the bax/bcl-2 ratio (Figure 4A). Oral treatment of YE reduced the bax/bcl-2 ratio in CS-exposed mouse alveolar tissues. The tumor suppressor p53 is known to directly activate bax and mediate mitochondrial membrane permeabilization and apoptosis [31]. As expected, the activation of p53 was enhanced in CS-loaded lung tissues, which was retarded by supplementing 25–100 mg/kg YE to mice (Figure 4B). In addition, caspase-9 and its downstream executioner caspase-3 responsible for executing cell death were upregulated in lung tissues by exposure to CS (Figure 4C). In contrast, YE highly attenuated the activation of these caspases in lung tissues. 

The reciprocal interactions among ROS, airway inflammation, and alveolar cell death play crucial role in the pathogenesis of COPD [32]. This study introduced DHE staining for ROS production in airways exposed to CS. DHE exhibits blue-fluorescence in the cytosol until oxidized, where it intercalates within the cell DNA, with subsequent staining of nuclei as a bright fluorescent red. There was a strong DHE staining in CS-loaded bronchioles and alveoli, indicating marked superoxide production by CS (Figure 4D). However, oral administration of 25–100 mg/kg YE highly attenuated ROS production in bronchiolar and alveolar tissues of CS-challenged mice.

### 3.4. Suppressive Effects of YE on CSE-Loaded Alveolar Apoptotic Injury

**Figure 5:**
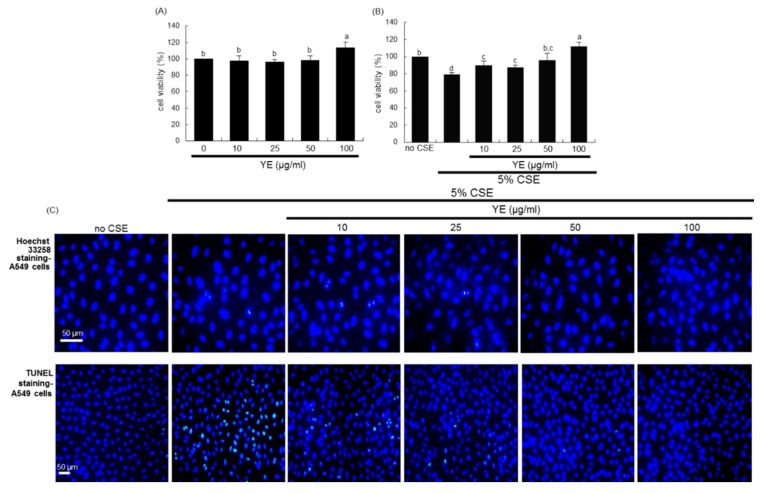
Viability of alveolar epithelial A549 cells and effects of dried yeast extracts (YE) on alveolar apoptosis. Alveolar epithelial cells were incubated in media containing 5% cigarette smoke extract (CSE) in the absence and presence of 10–100 μg/mL YE for up to 24 h. A549 cell viability (mean ± SEM, *n* = 5) was measured by using MTT assay and expressed as percent cell survival relative to untreated controls (**A**,**B**). Values in respective bar graphs not sharing a same small letter indicate a significant difference at *p* < 0.05. Nuclear staining was done with Hoechst 33258 dye for the detection of apoptotic cells (**C**). A transferase dUTP nick end labeling (TUNEL) assay was conducted to detect DNA fragmentation of apoptotic A549 cells and nuclear staining was accomplished with DAPI (**C**). Representative microphotographs were obtained by fluorescence microscopy. Scale bars = 50 μm.

This study further explored how CS evoked alveolar damage in mice and how YE protected alveoli from CS. The treatment of A549 cells with 10–100 μg/mL YE did not cause cytotoxicity for 24 h (Figure 5A). When 5% CSE was applied to alveolar epithelial A549 cells for 24 h, the viability dropped to below 20% (Figure 5B). When 5% CSE-loaded A549 cells were supplemented with ≥10 μg/mL YE for 24 h, the viability was highly boosted (Figure 5B). On the other hand, Hoechst 33258 nuclear staining and TUNEL staining showed that 5% CSE resulted in nuclear condensation and DNA fragmentation of A549 cells in an apoptotic manner (Figure 5C). The apoptotic cell death by CSE was significantly curtailed in YE-added alveolar cells. Accordingly, CS-induced alveolar emphysema may be ascribed to its apoptotic death of alveolar cells.

### 3.5. Inhibition of Airway Inflammation of OVA-Exposed Airways by YE

**Figure 6:**
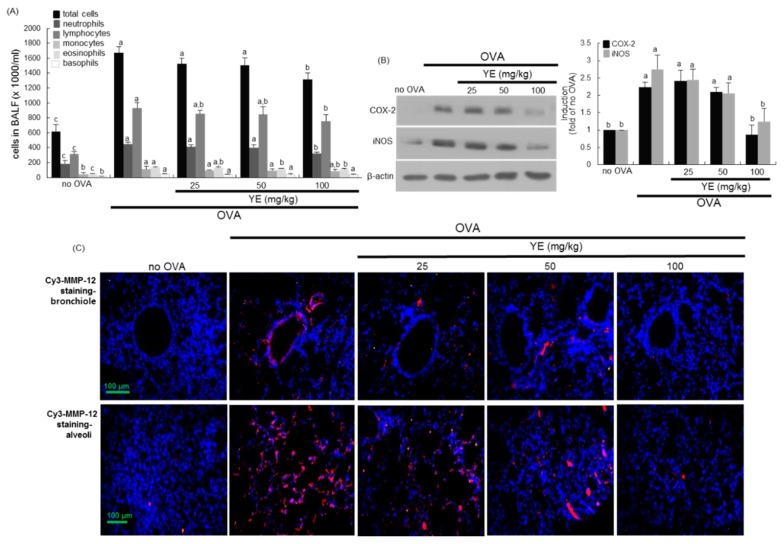
Suppressive effects of dried yeast extract (YE) on airway inflammation and induction of airway target proteins in ovalbumin (OVA) inhalation-challenged mouse lungs. OVA-sensitized mice were orally administrated with 25–100 mg/kg YE. Cells in BALF were counted using a Hemavet HV950 Multispecies Hematologic Analyzer (**A**). Lung tissue extracts were subject to Western blot with a primary antibody against COX-2 and iNOS (**B**). β-Actin protein was used as an internal control. The bar graphs (mean ± SEM, *n* = 3) represent quantitative results of the left bands obtained from a densitometer. Values in respective bar graphs not sharing a same small letter indicate a significant difference at *p* < 0.05 Immunohistofluorescence analysis was done in tissues of small airways and alveoli of OA-challenged mice (**C**). The MMP-12 localization was identified as Cy3-red staining in mouse airways exposed to OVA. Nuclear staining was done with DAPI (blue). Each photograph is representative of four mice. Scale bars = 100 μm.

This study investigated that YE inhibited allergic airway inflammation evoked by OVA in mouse airways. When mice underwent OVA inhalation, total leukocyte number in BALF was highly elevated by ≈2.5-fold (Figure 6A). The OVA inhalation prompted neutrophilic and eosinophilic inflammation in BALF, while oral administration of 25–100 mg/kg YE reduced OVA-induced leukocytosis of neutrophils and lymphocytes (Figure 6A). In addition, YE diminished eosinophilic inflammation elevated in OVA-exposed mice (Figure 6A).

The tissue levels of inflammatory COX-2 and iNOS were enhanced in OVA inhalation-experienced mouse lungs in a similar manner to the CS challenge (Figure 6B). However, the YE supply attenuated the lung tissue induction of these proteins promoted by OVA (Figure 6B). Accordingly, YE may alleviate OVA inhalation-induced allergic inflammation in airways. In addition, this study examined whether OVA induced pulmonary emphysema in mice. The Cy3-immunostaining revealed that OVA promoted the MMP-12 expression in mouse bronchioles and alveoli (Figure 6C). It should be noted that the MMP-12 induction by OVA inhalation was less than that of CS challenge. Nevertheless, YE curtailed the Cy3-MMP-12 staining in airways, indicating that YE abrogated pulmonary emphysema due to OVA (Figure 6C).

### 3.6. Blockade of LPS-Triggered Airway Inflammation by YE

**Figure 7:**
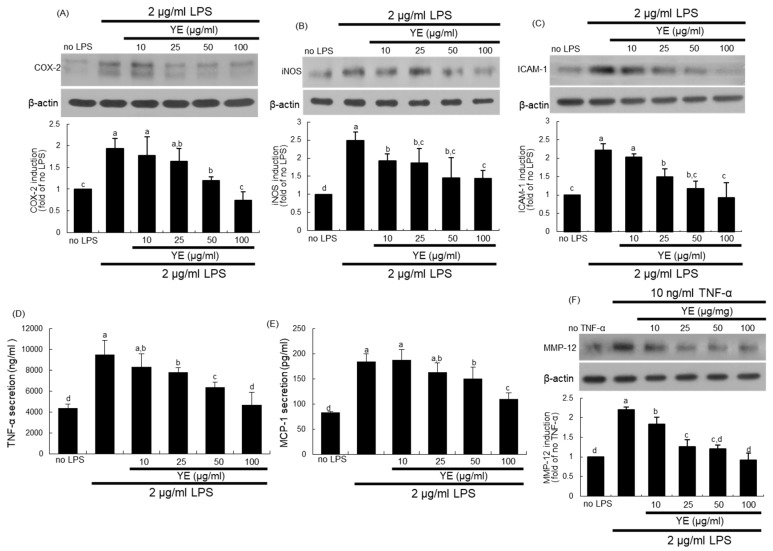
Blockade of alveolar inflammation by dried yeast extracts (YE) in lipopolysaccharide (LPS)-exposed A549 cells. Alveolar epithelial cells were incubated in media containing 2 μg/mL LPS or 10 ng/ml TNF-α in the absence and presence of 10–100 μg/mL YE for up to 24 h. Cell lysates were prepared for Western blot analysis with a primary antibody against COX-2, iNOS, ICAM-1, or MMP-12 (**A**–**C**,**F**). β-Actin protein was used as an internal control. The bar graphs (mean ± SEM, *n* = 3) represent quantitative results of the upper bands obtained from a densitometer. Alveolar secretion of TNF-α and MCP-1 was measured by using ELISA kits (**D**,**E**). Values in respective bar graphs not sharing a same small letter indicate a significant difference at *p* < 0.05.

The endotoxin LPS stimulated alveolar inflammation through the induction of COX-2, iNOS, and ICAM-1 in A549 cells (Figure 7A–C). In addition, LPS prompted the secretion of pro-inflammatory cytokines of TNF-α and MCP-1 from alveolar epithelial cells (Figure 7D,E). When LPS-loaded alveolar cells were treated with 10–100 μg/mL YE, such induction and secretion of these inflammatory proteins were reduced (Figure 7A–E). 

This study further examined whether pro-inflammatory TNF-α produced by alveolar cells might be involved in evoking alveolar emphysema by pathological stimuli. When TNF-α was treated to A549 cells, the MMP-12 protein was highly induced (Figure 7F). In contrast, ≥10 μg/mL YE blunted its induction in TNF-α-experienced alveolar cells. Thus, one can speculate that airway inflammation may be a contributor to pulmonary emphysema.

**Figure 8:**
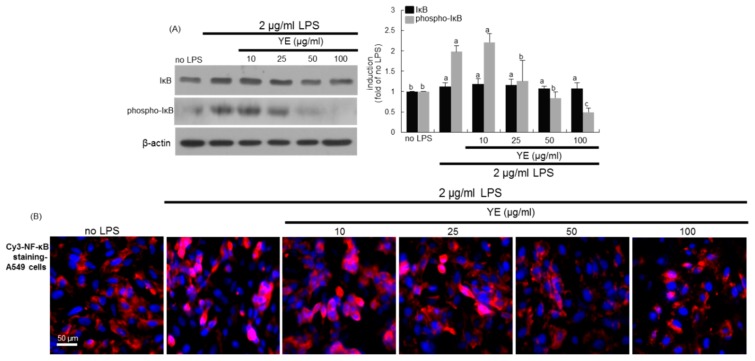
Involvement of NF-κB signaling in lipopolysaccharide (LPS)-induced alveolar inflammation and blockade by dried yeast extracts (YE). Alveolar epithelial cells were incubated in media containing 2 μg/mL LPS in the absence and presence of 10–100 μg/mL YE for up to 24 h. Cell lysates were prepared for Western blot analysis with a primary antibody against IκB and phospho-IκB (**A**). β-Actin protein was used as an internal control. The bar graphs (mean ± SEM, *n* = 3) represent quantitative results of the left bands obtained from a densitometer. Values in respective bar graphs not sharing a same small letter indicate a significant difference at *P* < 0.05. Immunocytofluorescence analysis was done in LPS-treated A549 alveolar epithelial cells (**B**). The NF-κB localization was identified as Cy3-red staining in cells exposed to LPS. Nuclear staining was done with DAPI (blue). Each photograph is representative of stained cells (*n* = 4). Scale bar = 50 μm.

It has been reported that sustained activation of NF-κB pathway links airway inflammation and COPD, which provides its potential as target for treatment of asthma and COPD [33]. LPS highly increased IκB phosphorylation of A549 cells, leading to induction of nuclear translocation of NF-κB (Figure 8A). The treatment of ≥10 μg/mL YE retarded its phosphorylation. Consistently, the Cy3-NF-κB staining supported the Western blot data showing nuclear translocation of NF-κB that was inhibited by YE (Figure 8B).

## 4. Discussion

Eight major findings were extracted from this study. (1) Oral administration of ≥25 mg/kg YE markedly reduced leukocytosis of neutrophils and eosinophils as well as lymphocytes and monocytes in the BALF of CS-exposed mice. (2) YE reduced the induction of these inflammatory proteins of COX-2, iNOS, and ICAM-1 together with reduction of the pathological alterations and oxidative stress in small airways and alveoli of CS-challenged mice. (3) The treatment with 25–100 mg/kg YE blunted the MMP-12 expression and emphysematous damage of airways through diminution of bax/bcl-2 ratio and inactivation of p53 and caspases in CS-exposed mouse lungs. (4) In 5% CSE-loaded A549 cells supplemented with 10–100 μg/mL YE the apoptotic cell death was significantly attenuated. (5) The OVA inhalation highly elevated total leukocyte number in BALF, while the YE treatment diminished OVA-triggered neutrophilic and eosinophilic inflammation. (6) The tissue levels of COX-2, iNOS, and MMP-12 were enhanced in OVA-exposed mouse lungs, which was curtailed by the supply of 25–100 mg/kg YE. (7) Treatment of 10–100 g/mL YE abrogated the induction of COX-2, iNOS, and ICAM-1 and the secretion of TNF-α and MCP-1 with blockade of NF-κB signaling in LPS-loaded alveolar cells. (8) The MMP-12 induction by pro-inflammatory TNF-α was blunted in YE-treated alveolar cells. Accordingly, CS- and OVA inhalation-induced pulmonary oxidative stress and inflammation may contribute to airway tissue destruction and emphysema. YE may inhibit emphysema and alveolar cell loss in airways and alveoli encountered in the oxidative and inflammatory milieu. Therefore, anti-inflammatory and antioxidant YE had a potential benefit in treating pulmonary diseases of COPD and asthma.

COPD is a chronic inflammatory lung disease with bronchial airflow impairment and obstruction caused by long-term exposure to irritating risk factors such as cigarette smoking and biomass fuel dust exposure [1,2]. The most common disorders involved in COPD are chronic bronchitis inflamed in the lining of the bronchial tubes and emphysema liable to the alveoli [5,6]. As expected, this study showed that CS and allergic OVA enhanced the lung induction of COX-2, iNOS, and ICAM-1 responsible for lung inflammation along with marked leukocytosis in BALF. Chronic lung inflammation involves the infiltration of inflammatory cells such as neutrophils, macrophages, and lymphocytes into the small airways of COPD [1,6]. Similarly, the exposure to CS stimulates the recruitment of inflammatory cells into the airways and elicits immune responses [14]. This study revealed that the exposure of both CS and OVA to mice evoked eosinophilic allergic inflammation in airways. The challenge of LPS to alveolar cells induced lung inflammation entailing the secretion of MCP-1 and TNF-α through activating NF-κB-responsive mechanism(s). Several studies highlight the role of NF-κB signaling in these two important inflammatory lung diseases of asthma and COPD [33,34]. The definite mechanisms underlying bronchiolar and alveolar inflammation are yet unsolved in COPD. However, to alleviate airway inflammation would be a primary therapeutic option for chronic bronchitis and COPD [12,13].

Emphysema is characterized by alveolar destruction with a loss of alveolar integrity and marked airspace enlargement [14,16]. This study showed that CS enhanced the MMP-12 induction in bronchiolar airways and alveoli, indicating that CS resulted in pulmonary emphysematous injury. Several promising mechanisms including oxidative stress and protease/anti-protease imbalance have been suggested for the small airway/alveolar destruction and structural changes, which may provide major therapeutic targets in COPD [10,11]. An activation of aberrant inflammation in bronchioles and alveoli lead to pathological alterations including loss of alveolar integrity [4]. Consistently, the pro-inflammatory TNF-α released by LPS promoted alveolar MMP-12 expression. Accordingly, airway inflammation due to OVA and CS may be a major contributor in the pathogenesis of emphysema. Oxidative stress contributes to cause alveolar emphysema through activating transcription of pro-inflammatory cytokines [13,17]. Several antioxidant natural compounds of epigallocatechin gallate and glycyrrhizin ameliorate oxidative renal injury and experimental acute pancreatitis in rats [35,36]. A growing body of evidence reports that CS as a risk factor in COPD is closely associated with increased oxidative stress [37,38]. Smoke may accelerate apoptosis of structural cells in the lung by means of oxidative stress, which might possibly be an important upstream event involved in the development of COPD and pulmonary emphysema [39,40]. In this study the exposure of CS to mice markedly enhanced apoptotic mediators of lung tissues as well as the ROS formation in alveoli. Another mechanism involved in the development of COPD is imbalance between proteolytic and anti-proteolytic activity [17,18,39]. This study found that inflammatory CS and OVA elicited proteolytic MMP-12 expression, resulting in the destruction of lung tissues. However, the emphasis on alveolar matrix destruction by the combination of inflammation, oxidative stress, and excessive proteolysis has failed to fully account for the COPD-specific mechanisms [40].

There is a need for safe and effective treatments that prevent these pulmonary diseases and have beneficial impacts on the course of COPD and asthma. Diverse molecular therapeutic targets for emphysema have been proposed from the identification of cellular and molecular mechanisms of the pathogenesis of COPD and asthma [12,16,39]. The pharmacological use of inflammatory mediator-targeted therapeutic agents in patients of COPD and asthma depends on clinical phenotypes and pathophysiological mechanisms [41]. Novel anti-inflammatory agents targeting lung inflammation include inhaled corticosteroids and β-adrenergic receptor agonists, phosphodiesterase-4 inhibitors, macrolides, and statins [14,19,20]. These agents can alter COPD-specific mechanisms involved in inflammation, mucin hypersecretion, and tissue destruction [14,19]. Asthmatics with eosinophilic inflammation but not with neutrophilic inflammation respond better to corticosteroids [41]. The choice of the optimal treatment In COPD and asthma should be based on the underlying immunopathology [15]. Although therapeutic approaches aim to target the chronic neutrophilic inflammation in COPD, targeting the underlying causes of the pulmonary neutrophilia such as smoking and oxidative stress might be more promising strategies. Due to the unique interplay between oxidative stress and pathogenesis of COPD, oxidative stress represents a novel target for the treatment of COPD and therapeutic opportunity with antioxidants arising [42]. 

Numerous therapeutic strategies with naturally occurring bioactive compounds that have mostly proven to be safe are currently under development for treating COPD and asthma [21]. Evidence suggests that the anti-inflammatory and anti-oxidative roles of some of the existing natural agents have potential values in the treatment of inflammatory lung diseases [21,43]. The dietary natural polyphenolic compounds of kaempferol and astragalin antagonized airway epithelial apoptosis and fibrosis and airway thickening along with pulmonary inflammation in OVA-inhaled mice [27,28]. Flavonoids rich in fruits and vegetables show their beneficial effects in asthmatic animal models [44]. Several studies demonstrate potential roles of natural compounds for the treatment of COPD [22,26]. Dietary polyphenols of curcumin, resveratrol, green tea catechins, quercetin, sulforaphane, and lycopene that possess anti-oxidative activities can influence various COPD-specific mechanistic aspects for the treatment and management of COPD [25]. Our recent study showed that dietary oleuropein rich in olive blunted pulmonary inflammation and alveolar destruction led to emphysema [29]. Oleuropein also blocked the infiltration of inflammatory and allergic cells into airways in CS-challenged mice. Our recent study found that YE inhibited bronchial epithelial eosinophilia and mucus-secreting goblet cell hyperplasia in OVA-exposed mice [30]. The present study revealed that YE blocked bronchiolar and alveolar inflammation and subsequent pulmonary emphysema in CS- and OVA-challenged COPD models, in which YE abrogated chronic inflammation, MMP-12 proteolytic activity, oxidative stress, and apoptosis of structural alveolar cells in the airways that might possibly be an important upstream event in the pathogenesis of COPD.

## 5. Conclusions

This study investigated that YE counteracted pulmonary emphysema due to the CS challenge and OVA inhalation through blocking pulmonary inflammation. Oral administration of YE diminished CS-elicited induction of pro-inflammatory COX-2, iNOS, and ICAM-1 in airways and leukocytosis in BALF. YE antagonized CS exposure-induced lung tissue damage and emphysematous destruction of proteolytic MMP-12 through inhibiting ROS-triggered apoptosis of alveolar cells due to CSE. In addition, oral supplementation of YE inhibited OVA-prompted pulmonary asthmatic inflammation and alveolar destruction concomitantly with reduced leukocytosis in BALF. Furthermore, YE suppressed LPS-induced alveolar inflammation of TNF-α and MCP-1 via activation of NF-κB signaling. Thus, YE may have a potential benefit in treating pulmonary diseases of COPD and asthma through inhibiting oxidative stress, pulmonary inflammation, and subsequent emphysematous damage. Although YE may serve as an antioxidant and modulator against airway inflammation and alveolar emphysema due to CS and OVA, its dietary role in COPD and asthma remains unclear. Further validation is required to clarify whether an appropriate intake of YE may constitute a dietary treatment for a therapeutically preventive strategy for COPD.

## Abbreviations

COPD	chronic obstructive pulmonary disease
COX-2	cyclooxygenase-2
CS	cigarette smoke
CSE	cigarette smoke extracts
YE	yeast extracts
IL	interleukin
iNOS	inducible nitric oxide synthase
LPS	lipopolysaccharide
MCP-1	monocyte chemoattractant protein-1
MMP-12	matrix metalloproteinase-12
NF-κB	nuclear factor-κB
OVA	ovalbumin
ROS	reactive oxygen species
TNF-α	tumor necrosis factor-α
